# Glucocorticoid-induced enhancement of extinction—from animal models to clinical trials

**DOI:** 10.1007/s00213-018-5116-0

**Published:** 2019-01-04

**Authors:** Dominique de Quervain, Oliver T. Wolf, Benno Roozendaal

**Affiliations:** 1 Transfaculty Research Platform, University of Basel, CH-4055 Basel, Switzerland; 2 Division of Cognitive Neuroscience, Department of Psychology, University of Basel, CH-4055 Basel, Switzerland; 3 University Psychiatric Clinics, University of Basel, CH-4012 Basel, Switzerland; 4 Department of Cognitive Psychology, Faculty of Psychology, Ruhr University Bochum, Bochum, Germany; 5 Department of Cognitive Neuroscience, Radboud University Medical Center, 6500 HB Nijmegen, The Netherlands; 6 Donders Institute for Brain, Cognition and Behaviour, Radboud University Nijmegen, 6525 EN Nijmegen, The Netherlands

**Keywords:** Glucocorticoids, Memory, Fear-related disorders, Clinical trials

## Abstract

Extensive evidence from both animal model and human research indicates that glucocorticoid hormones are crucially involved in modulating memory performance. Glucocorticoids, which are released during stressful or emotionally arousing experiences, enhance the consolidation of new memories, including extinction memory, but reduce the retrieval of previously stored memories. These memory-modulating properties of glucocorticoids have recently received considerable interest for translational purposes because strong aversive memories lie at the core of several fear-related disorders, including post-traumatic stress disorder and phobias. Moreover, exposure-based psychological treatment of these disorders relies on successful fear extinction. In this review, we argue that glucocorticoid-based interventions facilitate fear extinction by reducing the retrieval of aversive memories and enhancing the consolidation of extinction memories. Several clinical trials have already indicated that glucocorticoids might be indeed helpful in the treatment of fear-related disorders.

## Introduction

Stress mediators, including hormones, peptides, and neurotransmitters, promote the organism’s ability to cope with stress by acting on target systems in the periphery but also by exerting numerous effects on the brain (Joëls and Baram 2009). In addition to preparing an individual for the acute consequences of dangerous or threatening situations (i.e., fight-flight response) and the return to homeostasis, an important function of the stress response is to induce long-term adaptive responses, including influences on learning and memory (Roozendaal and McGaugh 2011). Notably, stressful and emotionally arousing events are typically remembered better than ordinary events (McGaugh 2003). By contrast, memory retrieval can be hampered during stressful and emotionally arousing conditions (de Quervain et al. 2009, 2017; Wolf 2017).

Extensive evidence from studies in animals have indicated that glucocorticoid hormones, in concert with many other stress mediators, are crucially involved in mediating the modulatory effects of stress on both the consolidation and retrieval of memory (Quirarte et al. 1997; de Quervain et al. 1998; Roozendaal et al. 2009). Furthermore, glucocorticoids are known to interact with arousal-induced noradrenergic activity to selectively modulate memory of emotionally arousing information or during emotionally arousing test situations (de Quervain et al. 2009). Importantly, these modulatory effects of glucocorticoids on emotional memory processes have also been found in studies with healthy humans (de Quervain et al. 2009; Wolf 2009; Schwabe et al. 2012).

It seems highly adaptive to have such biological processes that enable the significance of events to regulate their remembrance (McGaugh 2003; de Quervain et al. 2009). However, in case of extremely aversive events, overly traumatic or fearful memories may contribute to the development and symptoms of fear-related disorders, such as post-traumatic stress disorder (PTSD) and phobias. Current options for treating fear-related disorders mainly consist of exposure-based psychotherapy, which is based on extinction of conditioned fear by re-exposure to trauma- or fear-related memories, and/or anxiety-reducing and antidepressant medications. Psychotherapeutic interventions are generally successful, especially in phobias, but treatment response is diverging and the return of fear is a well-known problem (Bandelow et al. 2007). Exposure therapy is therefore ineffective in a substantial subgroup of over 50% of patients (Bradley et al. 2005; Bandelow et al. 2007; Cusack et al. 2016). Also, current pharmacological treatments such as anxiolytic or antidepressant drugs are far from satisfactory, since many patients continue to have symptoms (Barton et al. 2014). Such treatments primarily relieve stress and anxiety symptoms (Ipser et al. 2006) but do not diminish the underlying aversive memory trace (Lin et al. 2016). Therefore, new therapeutic approaches are desperately needed.

A possible pharmacological approach to prevent PTSD after trauma exposure is to reduce the initial consolidation of memory of aversive events, for example by the use of opioids (Holbrook et al. 2010) or beta-adrenergic receptor blockers (Pitman et al. 2002). Another approach would be to diminish the excessive retrieval of aversive memories, thereby reducing the severity and/or frequency of experienced symptoms such as intrusions and nightmares. Inhibition of memory retrieval during the first days or weeks after a traumatic event may also counteract the progressive formation of an overly strong traumatic memory trace, thus having preventing effects with regard to the development of PTSD. A further approach might consist of aiding the extinction of the traumatic memory trace (a process that is often impaired in patients with fear-related disorders) (Wessa and Flor 2007). This approach might be particularly well suited if the drug treatment is combined with exposure therapy in a timed manner to boost extinction and improve the long-term outcome of exposure therapy.

**Fig. 1:**
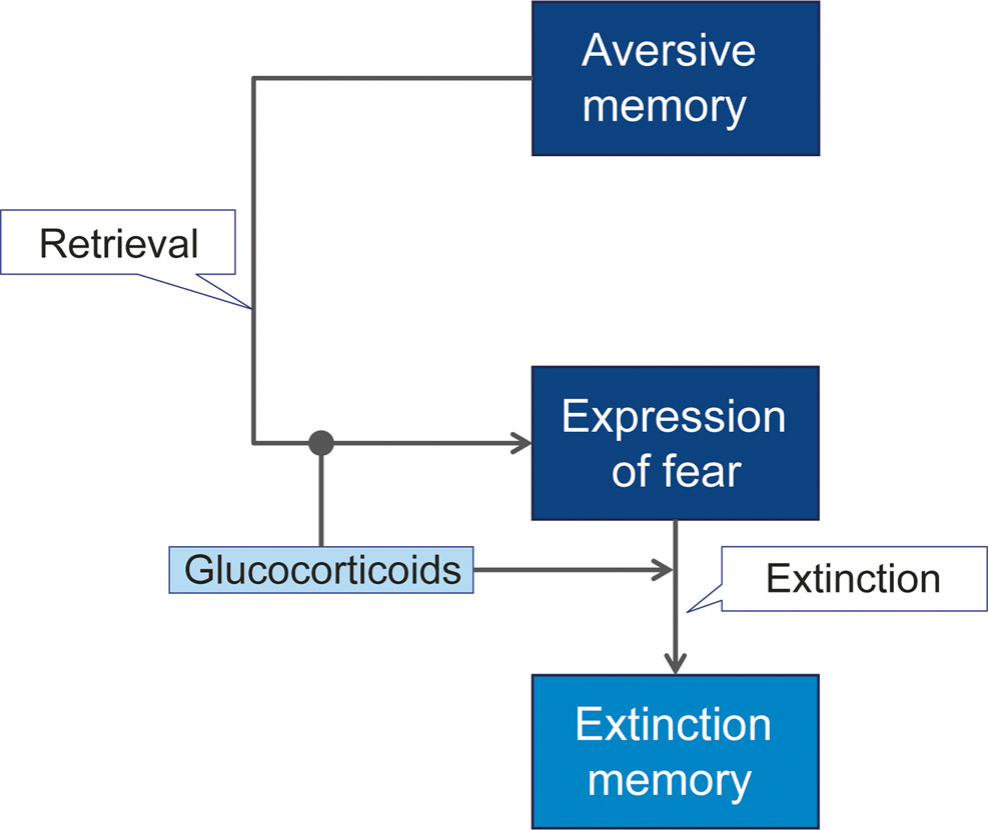
Glucocorticoid-induced enhancement of extinction. According to this model, glucocorticoids reduce the retrieval of aversive memories and thus curtail the expression of fear. A reduction of aversive memories may also support fear extinction: Experiencing reduced fear in otherwise fearful situations is likely to support fear extinction processes by promoting non-fearful, corrective experiences. Further, glucocorticoids enhance the consolidation of extinction memory

Here, we reason that glucocorticoid treatment is of special interest for preventing and treating fear-related disorders because they can affect multiple memory processes, i.e., reduce the retrieval of aversive memories and enhance the consolidation of extinction memories, that synergistically contribute to a reduction of fear-related symptoms (Fig. 1).

## Stress, glucocorticoids, and memory

Stress leads to an activation of the hypothalamus-pituitary-adrenal (HPA) axis. Activation of the HPA axis triggers a cascade of events that induces the release of glucocorticoids (mainly cortisol in humans, corticosterone in rodents) from the adrenal cortex (Ulrich-Lai and Herman 2009). First, corticotropin-releasing factor (CRF) is released by the hypothalamic paraventricular nucleus into the portal system. CRF then induces the release of adrenocorticotropin from the adrenal pituitary gland, which subsequently stimulates the release of glucocorticoids from the adrenal cortex into the bloodstream (Smith and Vale 2006). In the periphery, glucocorticoids exert, for example, immunosuppressive actions and increases in blood glucose levels (Wajchenberg et al. 1984; Sapolsky et al. 2000; Kuo et al. 2015).

In the 1960s and 70s, it was first discovered that glucocorticoids can facilitate the extinction of fear-motivated behaviors (Bohus and Lissak 1968). Many subsequent studies reported both enhancing and impairing properties of glucocorticoids on cognitive performance (Flood et al. 1978; Beckwith et al. 1986; Luine et al. 1993; Arbel et al. 1994; Kirschbaum et al. 1996). More recent studies indicated that glucocorticoids can have opposite effects on distinct memory processes. There is now extensive evidence for the view that glucocorticoids enhance the consolidation of memory of new information, including extinction memory, but impair the retrieval of already stored information (de Quervain et al. 2009; Schwabe et al. 2012). Most of these glucocorticoid effects on specific memory functions have been investigated in conditions with acute elevations of glucocorticoid levels, such as by an acute stressor or single glucocorticoid administration. Although clinical conditions with chronically elevated glucocorticoid levels are usually associated with impaired cognitive performance (Sapolsky 2000; McEwen 2001), it became clear that glucocorticoid administration even under such chronic stress conditions often exerts opposite effects on consolidation and retrieval processes. For example, comparable to the memory effects in acute conditions, a single glucocorticoid administration to patients with PTSD or phobias (which are chronic stress conditions) reduces the recall, but enhances the extinction, of fear memories (de Quervain et al. 2009). Furthermore, acute glucocorticoid administration also impairs retrieval processes in patients who have chronically elevated glucocorticoid levels (as a result of medication) (Coluccia et al. 2008). In the following sections, we discuss acute glucocorticoid effects on different memory processes.

### Consolidation

Memory consolidation refers to a process by which a fragile short-term memory trace is transferred into stable long-term memory (McGaugh 2000). However, not all information is equally well transferred into long-term memory. In fact, it is well recognized that especially emotionally arousing (pleasant or unpleasant) life events are remembered better than neutral events, even after a long period of time (McGaugh 2003). There is compelling evidence from studies in both animals and humans that glucocorticoids are crucially involved in regulating the consolidation of memory processes (Roozendaal 2000; McGaugh and Roozendaal 2002; Het et al. 2005; Sandi and Pinelo-Nava 2007; de Quervain et al. 2009; Roozendaal et al. 2009; Schwabe et al. 2012; de Quervain et al. 2017). Blockade of glucocorticoid production with the synthesis inhibitor metyrapone impairs consolidation processes in both animals and humans (Cordero et al. 2002; Maheu et al. 2004). In contrast, acute systemic glucocorticoid administration enhances long-term memory when given either before (Sandi and Rose 1994; Buchanan and Lovallo 2001; Abercrombie et al. 2003) or shortly after a training experience (Flood et al. 1978; Sandi and Rose 1994; Roozendaal and McGaugh 1996; Roozendaal et al. 1999a). Glucocorticoid manipulations applied before learning may affect both encoding and consolidation processes. Human work has shown that administration of glucocorticoids might affect the encoding of memory by having an influence on both sensory (Miller et al. 2015) and attentional processes (Putman and Roelofs 2011; Hermans et al. 2014). Glucocorticoid effects on memory consolidation follow an inverted U-shaped dose-response relationship: Moderate doses enhance consolidation processes, whereas lower or higher doses are typically less effective or even induce memory impairment (Roozendaal et al. 1999b; Andreano and Cahill 2006).

Some evidence indicates that stress effects on memory consolidation are more pronounced in men than in women (Andreano and Cahill 2006; Preuss and Wolf 2009; Cornelisse et al. 2011), possibly due to an interaction with sex hormones; in particular, hormonal contraceptives can raise cortisol-binding globulin (CBG) concentrations and therefore lead to a blunted free cortisol response (Kirschbaum et al. 1999) and reduce stress effects on memory (Preuss and Wolf 2009; Cornelisse et al. 2011; Merz and Wolf 2017). Consistent with these findings, no sex differences in memory effects were reported among people acutely dosed with exogenous cortisol (Buchanan and Lovallo 2001).

Evidence from several kinds of studies indicates that glucocorticoids interact with arousal-induced noradrenergic activity in influencing memory consolidation (Roozendaal and McGaugh 2011). For example, animal model studies have shown that glucocorticoid administration after footshock delivery in an inhibitory avoidance task rapidly augments noradrenaline levels within the basolateral amygdala (McReynolds et al. 2010). On the other hand, attenuation of noradrenergic signaling with a beta-adrenergic receptor antagonist administered systemically or directly into the basolateral amygdala blocks the enhancing effect of glucocorticoids on memory consolidation for emotionally arousing training experiences (Quirarte et al. 1997; Roozendaal et al. 2006). Further, glucocorticoid administration immediately after object recognition training enhances 24-h memory of emotionally aroused rats but not that of rats previously habituated to the training context in order to reduce novelty-induced emotional arousal (Okuda et al. 2004; Roozendaal et al. 2006). However, with such low-arousing conditions, pharmacological reinstatement of (nor)adrenergic activity by the administration of the noradrenergic stimulant yohimbine enables glucocorticoid-induced memory enhancement (Roozendaal et al. 2006). Human studies generally support the conclusion of animal experiments in that glucocorticoids enhance memory consolidation only when their activity is paralleled by emotional arousal (i.e. noradrenergic activity) (Kuhlmann and Wolf 2006a; Segal et al. 2014). Cortisol administered shortly before or after training selectively enhances long-term memory of emotionally arousing, but not of emotionally neutral, items (Buchanan and Lovallo 2001; Kuhlmann and Wolf 2006a). Moreover, a cold pressor stress in humans (i.e., placing the arm in ice water), a procedure that significantly elevates endogenous cortisol levels, enhances memory of emotionally arousing slides, but does not affect memory of emotionally neutral slides (Cahill et al. 2003; Preuss and Wolf 2009).

Glucocorticoid hormones are highly lipophilic (McEwen et al. 1979) and bind directly to mineralocorticoid receptors (MRs) and glucocorticoid receptors (GRs) in the brain (Reul and de Kloet 1985; Arriza et al. 1987). MRs have a high affinity for the natural steroids corticosterone, cortisol and aldosterone, whereas GRs have an approximately 10 times lower affinity for corticosterone and cortisol but show a high affinity for the synthetic ligand dexamethasone (Reul et al. 1987; Sutanto and de Kloet 1987). The memory-enhancing effects of glucocorticoids appear to involve the selective activation of the low-affinity GR (Oitzl and de Kloet 1992; Roozendaal and McGaugh 1997). Some studies, however, indicated that MR function, either alone or in conjunction with GRs, might also be implicated in mnemonic functions (Rimmele et al. 2013; Atucha et al. 2015; Hinkelmann et al. 2015), but in most cases, evidence for a direct influence on consolidation processes is lacking.

Glucocorticoids are known to act through intracellular and intranuclear receptors and can affect neuronal function through their ability to affect gene transcription (Datson et al. 2001). However, glucocorticoids also have various non-genomic actions on neuroplasticity and memory, through a membrane-associated variant (or variants) of the steroid receptor (Johnson et al. 2005; Barsegyan et al. 2010; Riedemann et al. 2010; Roozendaal et al. 2010; Lee et al. 2011). Activation of these membrane steroid receptors results in effects such as a rapid increase in glutamate-release probability from presynaptic sites (Karst et al. 2005) and rapid insertion of AMPA-receptor subunits into postsynaptic membranes (Conboy and Sandi 2010; Krugers et al. 2010). Several experimental findings have shown that glucocorticoid effects on increasing noradrenergic signaling also have an onset that is too fast to be mediated via transcriptional regulation in the nucleus and likely involve a rapid, non-genomic mode of action. Glucocorticoids and noradrenaline signaling mechanisms might act synergistically to rapidly enhance AMPA-receptor function (Zhou et al. 2012) as well as to influence several other molecular events—for example, such interactions may induce rapid phosphorylation of the transcription factor cAMP-responsive element-binding protein (CREB) which, after binding to CREB-binding protein (CBP), promotes associated epigenetic mechanisms such as histone acetylation (Roozendaal et al. 2010; Chen et al. 2012). A recent study indicated that a moderate dose of glucocorticoids combined with noradrenergic stimulation caused a transient enhancement of glutamatergic transmission within the basolateral amygdala, but that this time window of excitability was extended in conditions mimicking severe stress (Karst and Joëls 2016). These genomic and non-genomic glucocorticoid actions may ultimately, and collectively, result in neuroplasticity and structural changes, e.g., via modifications of cell-adhesion molecules, and strengthen cell-cell interactions (Sandi 2011).

**Fig. 2:**
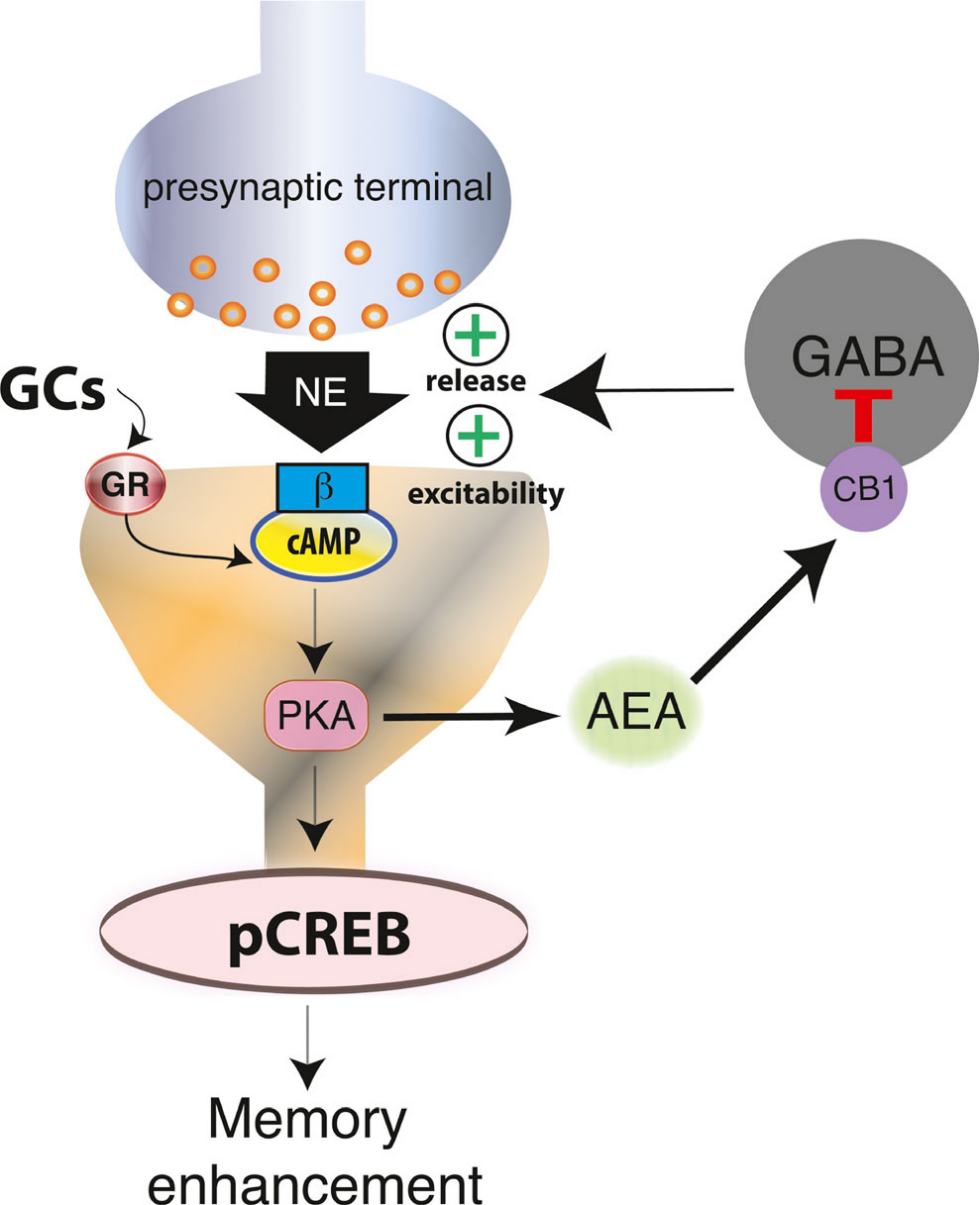
Role of the endocannabinoid system in regulating glucocorticoid effects on memory consolidation. Glucocorticoids, released during emotionally arousing situations, bind to a membrane-bound GR, and activate the intracellular cAMP/PKA signaling cascade. This triggers the release of endocannabinoids, particularly anandamide (AEA). Anandamide then activates CB1 receptors on GABAergic interneurons and thereby inhibits GABA release. This subsequently disinhibits norepinephrine (NE) release and increases the excitability of pyramidal neurons within the basolateral amygdala. This overall increases the sensitivity of basolateral amygdala neurons to the effects of norepinephrine and results in an increased activation of the cAMP (cyclic adenosine monophosphate) / PKA (protein kinase A) pathway and phosphorylation of the transcription factor CREB (cAMP response-element binding) protein. These stress hormone effects in the basolateral amygdala are required for enhancement of memory for emotionally arousing experiences by influencing information storage processes in other brain regions. Adapted from Atsak et al., Neuropsychopharmacology, 2015

Recent findings indicate that the actions of glucocorticoids on memory consolidation also involve intriguing rapid signaling interactions with the endocannabinoid system (Campolongo et al. 2009). Endogenous ligands for cannabinoid receptors, endocannabinoids, are synthesized on demand from lipid precursors in the postsynaptic membrane, and serve as retrograde messengers at both excitatory and inhibitory neurotransmission (Kano 2014) and therefore are key players in fine-tuning neural activity (Ohno-Shosaku and Kano 2014). The endocannabinoid system is closely linked to the glucocorticoid stress system (Hill et al. 2010; Atsak et al. 2012b; Morena et al. 2015), and emerged as a key modulator of the stress response (Hill et al. 2010; Morena et al. 2015), emotion regulation (Evanson et al. 2010; Marco and Laviola 2012) and emotional memory (Morena et al. 2016). Most importantly, it was found that a cannabinoid type-1 (CB1) receptor antagonist administered into the basolateral amygdala blocks the ability of glucocorticoids to facilitate aversive memory consolidation (Campolongo et al. 2009; Atsak et al. 2015). Acute glucocorticoid administration or training on an emotionally arousing task *rapidly* increases endocannabinoid levels in the amygdala as well as in other brain regions such as the hippocampus and prefrontal cortex (Adams et al. 2003; Hill et al. 2010; Morena et al. 2014). The findings suggest that glucocorticoids might bind to a membrane-located receptor that activates a G protein-coupled signaling cascade inducing endocannabinoid synthesis (Di et al. 2016). Endocannabinoid ligands then could diffuse and bind to presynaptic CB1 receptors. What the endocannabinoids will do next is unclear at this time. Possibly, endocannabinoids target local GABAergic terminals to inhibit GABA release onto noradrenergic terminals (Di et al. 2016), thus increasing the local release of noradrenaline (Fig. 2).

### Retrieval

Memory retrieval refers to the mental process of recollecting information from the past. In contrast to the enhancing effects of glucocorticoids on memory consolidation, stress exposure or glucocorticoid administration to rats or mice shortly before retention testing impairs the retrieval of memory (de Quervain et al. 1998). Although the vast majority of studies have investigated the effects of stress and glucocorticoids on the retrieval of hippocampus-dependent forms of memory in contextual fear conditioning and spatial water-maze tasks, some animal model studies have shown that stress exposure or glucocorticoid administration before retention testing also impairs the retrieval of cortex-dependent recognition memory (Barsegyan et al. 2015) and striatum-dependent stimulus-response associations (Atsak et al. 2016). These temporary effects of glucocorticoids on memory retrieval impairment (the effects dissipate when glucocorticoid levels have returned to baseline) are rapidly induced and do not seem to depend on gene transcription (Sajadi et al. 2006) and might selectively depend on the membrane GR (Chauveau et al. 2010) and being mediated via non-genomic actions (Roozendaal et al. 2004). Glucocorticoid-induced impairment of memory retrieval may help to suppress behaviors that are no more relevant or even maladaptive and a more opportune coping response is required (de Kloet et al. 1999). This mechanism is especially important in situations when the organism is forced to adapt to a changed environment.

Highly comparable to these findings in animal models, stress exposure or glucocorticoid administration also impairs retrieval processes in humans. A single administration of cortisone (at a dose resulting in high physiological cortisol levels) 1 h before retention testing impaired the recall of words learned 24 h earlier (de Quervain et al. 2000). Moreover, increased cortisol levels due to psychological stress have also been shown to impair declarative memory retrieval (Domes et al. 2004; Kuhlmann et al. 2005b; Buchanan et al. 2006; Shields et al. 2017). Glucocorticoid effects on memory retrieval are highly comparable to those seen in studies investigating memory consolidation in that the effects depend on emotional arousal. Specifically, it has been shown in studies in humans that the retrieval of emotionally arousing information is also particularly sensitive to impairment by glucocorticoids (de Quervain et al. 2000; Wolf et al. 2001; Buss et al. 2004; Het et al. 2005; Kuhlmann et al. 2005a, b; Smeets et al. 2005; Buchanan et al. 2006; Kuhlmann and Wolf 2006b; de Quervain et al. 2007; Smeets et al. 2008; Schwabe et al. 2009; Tollenaar et al. 2009). Glucocorticoid effects on memory retrieval also depend critically on noradrenergic activity within the brain (de Quervain et al. 2007; Schwabe et al. 2009). The influence of glucocorticoid–noradrenergic interactions on memory retrieval was further shown to also depend on the endocannabinoid system (Atsak et al. 2012a; Morena et al. 2015). Also comparable to memory consolidation, stress effects on memory retrieval are more prominent in men than in women who use hormonal contraceptives (Kuhlmann and Wolf 2005), suggesting possible interactions with sex hormones (Merz and Wolf 2017).

### Extinction

Extinction is a process in which conditioned responses to a stimulus previously paired with an aversive event diminish if the conditioned stimulus is presented repeatedly without the reinforcing stimulus (Quirk and Mueller 2008). Like other forms of learning, extinction learning is followed by a consolidation phase. Whereas the consolidation of extinction memory and that of new memory show partially distinct molecular and neuroanatomical profiles (e.g., different role of the prefrontal cortex) (Milad and Quirk 2012), glucocorticoids seem to play a similar role in both. Animal models have shown that glucocorticoid administration enhances the consolidation of extinction memory (Barrett and Gonzalez-Lima 2004; Cai et al. 2006; Yang et al. 2006; Blundell et al. 2011) whereas a suppression of glucocorticoid signaling impairs extinction consolidation (Bohus and Lissak 1968; Barrett and Gonzalez-Lima 2004; Yang et al. 2006; Blundell et al. 2011; Clay et al. 2011). More specifically, glucocorticoids administered either before or after extinction learning modify extinction processes of several types of fear memory, including auditory fear conditioning (Barrett and Gonzalez-Lima 2004), contextual fear conditioning (Cai et al. 2006; Blundell et al. 2011) and fear-potentiated startle (Yang et al. 2006), and in the predator stress paradigm (Clay et al. 2011). Furthermore, direct administration of the GR agonist dexamethasone into the amygdala prior to extinction training was found to enhance extinction memory (Yang et al. 2006).

**Fig. 3:**
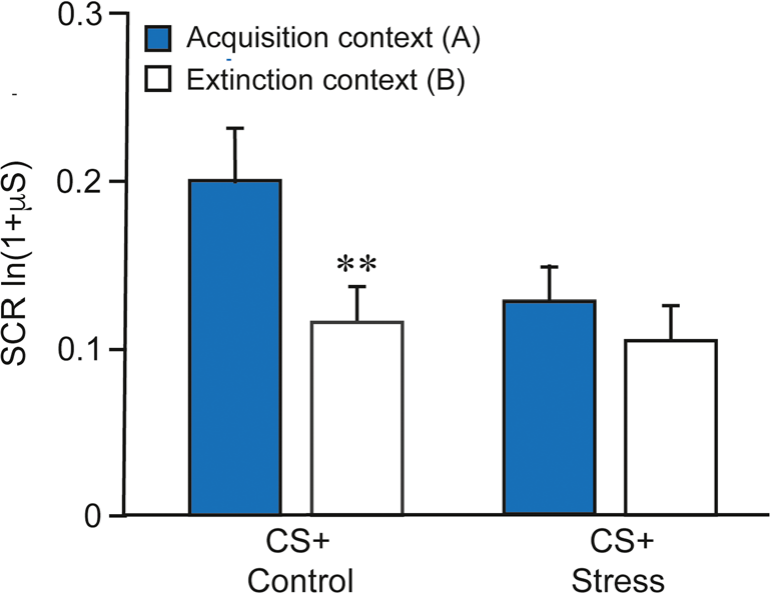
Stress before extinction learning reduced the return of fear in a renewal paradigm. The renewal test compared the mean skin conductance response (SCR) to the conditioned stimuli (CS+) in both contexts A (acquisition context) and B (extinction context). The control group (left panel; *n* = 20) showed renewal of the extinguished fear response (** *P* < .001: the response to the previously extinguished CS+ in context A is higher than in context B), the stress (socially evaluated cold pressor test 25–30 min before extinction learning) group (right panel; n = 20) showed no renewal. These results suggest a stronger and more generalized extinction memory in the stress group. Error bars represent SEM and thus between-subject variance. CS+, conditioned stimulus. Adapted from (Meir Drexler et al. 2018)

Current theories of extinction learning postulate that during extinction a safety memory trace is established since the stimulus is no longer followed by an aversive event. The ‘competition’ between the original fear memory trace and the safety memory trace acquired during extinction can explain the well-documented recovery phenomena of spontaneous recovery, reinstatement, and renewal (Vervliet et al. 2013). Extinction is thought to be more context dependent than the originally acquired fear memory (Bouton et al. 2006). In the laboratory, this context dependency can be studied using a renewal paradigm where acquisition takes place in context A, extinction learning in context B, and extinction retrieval is tested in both contexts A and B. Typically, the return of fear is stronger in the acquisition context (A). This mechanism might underlie the return of fear after a (seemingly) successful extinction-based therapy (e.g., when a patient again encounters a spider at home). In a series of human studies, the impact of stress on extinction and renewal was investigated using a contextual fear conditioning paradigm originally developed by Milad and colleagues (Milad et al. 2007). Stress (the Socially Evaluated Cold Pressor Test) induced 25–30 min before extinction learning enhanced the consolidation of extinction memory and made it less context dependent as evident by a reduced renewal effect (Meir Drexler et al. 2018) (Fig. 3). In contrast, stress induced immediately after extinction learning made the extinction memory more context dependent (Hamacher-Dang et al. 2015). Thus, from a clinical perspective, stress induction or cortisol administration should occur before extinction training in order to create a stronger and less context-dependent extinction memory trace. In a recent pharmacological functional MRI (fMRI) study (Merz et al. 2018), cortisol administered before extinction diminished activation of the amygdala-hippocampal neural network at the beginning of extinction and enhanced functional connectivity of the anterior parahippocampal gyrus with the ventromedial prefrontal cortex (vmPFC), a brain region crucially involved in extinction processes (Milad and Quirk 2002). These network alterations may underlie the blocking effects of cortisol on the retrieval of the initial fear memory and its combination with the beneficial effects on the consolidation of fear extinction memory (Nakataki et al. 2017).

The long-term efficacy of extinction-based therapies is not only determined by the initial extinction success, but also by the ability to retrieve the extinction memory when encountering the previously feared stimulus again (e.g., when meeting a spider at home in the cellar) (Quirk and Mueller 2008). In this situation, the initial fear memory trace and the inhibitory extinction memory trace have to compete and the ‘winner’ determines the actual behavioral response. Hence, the question arises how stress or glucocorticoids influence the retrieval of extinction memory. Two recent studies provided first evidence that acute stress can impair extinction retrieval in humans. In one study, a predictive learning task was used (Hamacher-Dang et al. 2013) while the other study used a classical fear conditioning paradigm (Raio et al. 2014). In both studies, stress was associated with a return of the originally learned behavior (or emotion). Supporting findings have been obtained in rodent models (Deschaux et al. 2013). However, conflicting findings have been reported as well (Merz et al. 2014). Similar findings have been reported after pharmacological glucocorticoid administration. In two independent fMRI studies, it was reported that cortisol administered before extinction recall testing induced a return of fear of the previously extinguished response (Kinner et al. 2016; Kinner et al. 2018). This was associated with reduced activity of the vmPFC (Kinner et al. 2016) and enhanced signaling in the amygdala (Kinner et al. 2018). Interestingly, this effect was absent in women using hormonal contraceptives, again pinpointing to a modulatory influence of sex hormones.

Taken together, the laboratory findings obtained so far suggest that glucocorticoids can facilitate extinction when given before extinction training (in line with the model proposed above (Fig. 1)). In contrast, elevated glucocorticoid concentrations at times of extinction retrieval might cause a return of fear by impairing extinction recall.

## Clinical implications

Based on the evidence from basic animal model and human studies reviewed above, glucocorticoids could be administered at different time points to reduce fear in clinical conditions. Glucocorticoids could be administered to diminish the retrieval of aversive memories, thereby reducing the expression of fear, such as of reexperiencing the traumatic event in PTSD. It is important to note that a reduced recall of aversive memories may also support fear extinction: Experiencing reduced fear in otherwise fearful situations is likely to support fear extinction processes by promoting non-fearful, corrective experiences (de Quervain et al. 2009). Further, glucocorticoid administration could be used to support the consolidation of extinction memories in patients who undergo extinction-based psychotherapy. These glucocorticoid signaling-based intervention strategies are illustrated in Fig. 1. This figure also illustrates the importance of the context (i.e. with or without concurrent extinction training), timing and duration of glucocorticoid administration (for a comprehensive review see Joëls et al. (2012). We now review clinical studies that used glucocorticoid signaling-based interventions to prevent or treat fear-related disorders (Table 1).

**Table 1:** Clinical trials with glucocorticoid-based interventions in fear-related disorders

Drug	Design	Timing and duration	Memory phase exposed	Outcome	Refs
Treatment of PTSD					
Cort	DB, PC, CO	Daily for 30 days	Retrieval	↓ Intrusions while under treatment	Aerni et al. 2004
Cort	DB, PC, CO	Daily for 7 days	Retrieval	No change in intrusions while under treatment	Ludascher et al. 2015
Cort	RCT	Single dose after exposure	Extinction	↓ PTSD symptoms at 1 week	Suris et al. 2010
Cort	RCT	20 min before exposure therapy, on 8 days	Retrieval and extinction	↓ PTSD symptoms at 6 weeks	Yehuda et al. 2015
Prevention of PTSD					
Cort	RCT	Starting < 12 h after trauma, for 10 days	Consolidation and retrieval	↓ PTSD symptoms at 3 months	Delahanty et al. 2013
Cort	RCT	Starting < 6 h after trauma, for 6 days	Consolidation and retrieval	↓ PTSD incidence at 31 months	Schelling et al. 2001
Cort	RCT	Starting < 6 h after trauma, for 4 days	Consolidation and retrieval	↓ Stress scores at 6 months	Schelling et al. 2004
Cort	RCT	Starting < 6 h after trauma, for 4 days	Consolidation and retrieval	↓ Stress scores at 6 months	Weis et al. 2006
Cort	RCT	Single dose < 6 h after trauma	Consolidation	↓ PTSD incidence at 3 months	Zohar et al. 2011
Dex	RCT	Single intraoperative dose	Consolidation	No difference in PTSD incidence at 18 months	Kok et al. 2016
Treatment of social phobia					
Cort	RCT	Single dose 1 h before phobic stimulus	Retrieval	↓ Fear while under treatment	Soravia et al. 2006
Treatment of spider phobia					
Cort	RCT	1 h before phobic stimulus, on 4 days	Retrieval and extinction	↓ Fear while under treatment	Soravia et al. 2006
Cort	RCT	1 h before exposure therapy, on 2 days	Retrieval and extinction	↓ Fear at 1 month	Soravia et al. 2014
Treatment of phobia of heights					
Cort	RCT	1 h before exposure therapy, on 3 days	Retrieval and extinction	↓ Fear at 1 month	de Quervain et al. 2011

Only randomized controlled trials (RCT) or double-blind (DB), placebo-controlled (PC), cross-over (CO) trials are included. Cort: Cortisol; Dex: Dexamethasone. Adapted from (de Quervain et al. 2017)

### PTSD

In contrast to what could be expected from a stress-related disorder, PTSD is not characterized by higher glucocorticoid levels (Meewisse et al. 2007), but rather by an enhanced HPA-axis feedback (Yehuda 2002; Pitman et al. 2012), often resulting in lower circulating cortisol levels than found in healthy people (Yehuda et al. 1991; Meewisse et al. 2007). Low cortisol levels depend on several factors, including gender and type and onset of trauma (Meewisse et al. 2007) and may contribute to a hyper-retrieval of aversive memories, promoting reexperiencing symptoms of PTSD (de Quervain et al. 2009). In contrast, low glucocorticoid signaling at the time of initial traumatic memory formation should, at least theoretically, be favorable, considering the enhancing properties of glucocorticoids with regard to memory consolidation. Therefore, GR antagonists could be used to block the initial consolidation of a traumatic experience, serving as a secondary prevention of PTSD. However, so far there are no clinical data available regarding this approach.

Another approach aimed at diminishing symptoms of PTSD by reducing the retrieval of aversive memories has been investigated by two studies. The first study was a double-blind, placebo-controlled, cross-over study in three patients. This study reported that low-dose cortisol treatment (10 mg per day for 1 month) diminished re-experiencing symptoms, such as daytime recollections, intrusions, and nightmares, even beyond the treatment period (Aerni et al. 2004). The second study, which used a similar design but in a larger group of patients receiving various psychotropic medications (including serotonin- or noradrenaline-reuptake inhibitors), did not find beneficial effects of cortisol treatment (10 mg or 30 mg per day) on PTSD symptoms (Ludascher et al. 2015). If memory retrieval is reduced during the first days or weeks after a traumatic event, it may also help to counteract the formation of an overly strong memory trace: By inhibiting memory retrieval, cortisol may partly interrupt the vicious cycle of spontaneous retrieving, re-experiencing and reconsolidating traumatic memories in PTSD and, thereby, promote forgetting, a spontaneous process that occurs when memory is not reactivated. Furthermore, high cortisol levels at the time of confrontation with an aversive cue may facilitate the extinction of aversive memory. Two mechanisms may contribute to this facilitation: (i) because of the cortisol-induced reduction of memory retrieval, an aversive cue is no longer followed by the usual aversive memory retrieval and related clinical symptoms but, instead, becomes associated with a non-aversive experience, which is stored as extinction memory; (ii) glucocorticoids can facilitate the consolidation of memory of these corrective experiences.

Clinical trials investigating the effects of glucocorticoid treatment on extinction memory have found that such treatment indeed facilitates extinction processes (Aerni et al. 2004; Suris et al. 2010; Yehuda et al. 2010; Yehuda et al. 2015). In particular, a recent randomized, double-blind, placebo-controlled trial in 24 PTSD veterans reported that the administration of cortisol (30 mg) combined with exposure treatment improved treatment retention and outcome (Yehuda et al. 2015). Moreover, a significant treatment condition by responder status interaction for glucocorticoid sensitivity indicated that responders to cortisol augmentation had the highest pre-treatment glucocorticoid sensitivity that diminished over the course of treatment (Yehuda et al. 2015).

Several studies have used high-dose glucocorticoid administration for a longer time period (typically several days) in the aftermath of a traumatic event. Here, glucocorticoids likely affected several memory phases. These studies indicate that prolonged treatment with high doses of cortisol that started within 12 h after trauma reduces the risk for the development of later PTSD (Schelling et al. 2001, 2004; Weis et al. 2006; Delahanty et al. 2013). A potential mechanism might be that high doses of glucocorticoids—due to the inverted-U-shaped dose-response relationship for the effects of glucocorticoids on consolidation—may have resulted in an impairment of consolidation (Roozendaal et al. 1999b), and/or by a reduction of the retrieval of the traumatic memory and thereby interrupting the vicious cycle of retrieving, re-experiencing and reconsolidating aversive memories (de Quervain et al. 2009). These effects of glucocorticoid administration on reducing the risk for the development of PTSD are consistent with the findings of other studies indicating that the risk for PTSD is decreased by higher excretion of endogenous cortisol in the first hours after a traumatic event (McFarlane et al. 1997; Yehuda et al. 1998; Delahanty et al. 2000).

Recently, two systematic reviews suggested that the prolonged administration of glucocorticoids after a traumatic event is the most effective pharmacological intervention currently available for the prevention of PTSD (Amos et al. 2014; Sijbrandij et al. 2015). One review included seven randomized controlled trials investigating the efficacy of several pharmacological treatments (4 with cortisol, 3 with the beta-adrenergic receptor antagonist propranolol, one with the selective serotonin-reuptake inhibitor escitalopram and one with the benzodiazepine temazepam). The authors found that cortisol, but none of the other drugs, showed efficacy in reducing the risk for PTSD in adult patients (Amos et al. 2014). The other review included 5 placebo-controlled studies with cortisol and reported a large effect of cortisol in preventing PTSD (Sijbrandij et al. 2015). Currently, there are several ongoing trials investigating the effects of cortisol administration on the development of PTSD (NCT00855270 https://clinicaltrials.gov/ct2/show/NCT00855270?term=NCT00855270&rank=1),(NCT02402114 https://clinicaltrials.gov/ct2/show/NCT02402114?term=NCT02402114&rank=1) and on fear extinction in veterans with PTSD (NCT00674570 https://clinicaltrials.gov/ct2/show/NCT00674570?term=NCT00674570&rank=1).

Human genetic and epigenetic studies have found several alterations in the glucocorticoid system associated with PTSD risk. *NR3C1 alterations.* The BclI polymorphism, a C to G nucleotide change associated with receptor hypersensitivity to glucocorticoids and lower plasma cortisol levels, is a single nucleotide polymorphism of the GR gene (*NR3C1*, nuclear receptor subfamily 3, group C, member 1 (van Rossum et al. 2003). Studies in healthy humans have indicated that GG carriers (as compared to GC and CC carriers) of the BclI polymorphism show enhanced emotional memory (Ackermann et al. 2013), and increased incidence of traumatic memories in patients who underwent intensive care therapy (Hauer et al. 2011). Furthermore, increased expression of *NR3C1* in peripheral blood mononuclear cells has been found to be related to higher PTSD risk, which is in line with the enhanced GR feedback found in PTSD patients (van Zuiden et al. 2011). Moreover, there is evidence that these alterations are partly epigenetically controlled. Two recent studies found that methylation of the *NR3C1* promoter is inversely related with lifetime PTSD risk (Labonte et al. 2014; Yehuda et al. 2014). Further, a study in genocide survivors reported that decreased methylation at the *NGFIA* (nerve growth factor-induced protein A) binding site of the *NR3C1* promoter is related with increased traumatic memories and increased PTSD risk (Vukojevic et al. 2014). In support of the idea that methylation level of the GR gene might regulate memory processes, this study also found that decreased methylation at the *NGFIA* binding site of healthy individuals was associated with enhanced picture recognition memory and related brain activity. Together, these studies point to an epigenetic and genetic link between the predisposition to form strong aversive memories and the risk for PTSD. *FKBP5 alterations.* FKPB5 is known to act as a co-chaperone that modulates GR activity (Zannas et al. 2016). Common alleles of *FKPB5* have been related to differences in GR sensitivity, PTSD risk, and the incidence of intrusive memories of aversive photographs (Mehta et al. 2011; Cheung and Bryant 2015). Furthermore, allele-specific demethylation of *FKBP5* has been reported to mediate gene–childhood trauma interactions. Specifically, demethylation of *FKBP5* was associated with increased stress-dependent gene transcription, followed by a dysregulation of the HPA axis and cortisol levels (Klengel et al. 2013). *FKBP5* alleles may also influence exposure-based psychotherapy in PTSD (Wilker et al. 2014), and *FKBP5* allele-specific alterations in methylation have been associated with differential responses to psychological treatments for anxiety disorders (Roberts et al. 2015). Taken together, the findings indicating that genetic and epigenetic variations in the glucocorticoid system are associated with aversive and traumatic memory, the risk for PTSD and treatment response, help to better understand the basis of individual differences in risk or resilience for PTSD. It is important to note, however, that common genetic polymorphisms, which typically have small effect sizes, cannot be used for diagnostic and/or personalized treatment purposes. More research is needed to evaluate whether rare genetic variants or specific methylation events might be better suited for such purposes.

With regard to the importance of the endocannabinoid system, which as indicated interacts with glucocorticoids in regulating emotional memory (Campolongo et al. 2009), two studies have suggested that a polymorphism (rs1049353) of the CB1 receptor gene (*CNR1*) is associated with PTSD risk (Lu et al. 2008; Mota et al. 2015). Furthermore, stimulation of CB1 receptors promotes memory extinction (for review, see de Bitencourt et al. (2013), and first clinical evidence suggests that cannabinoids might be useful in the treatment of PTSD (Roitman et al. 2014; Jetly et al. 2015). Thus, there is now evidence from independent studies indicating that the glucocorticoid and endocannabinoid systems are involved in extinction memory and that these two systems might be promising targets for pharmacological intervention aimed at the prevention and/or treatment of PTSD. In particular, the evidence discussed above indicating that these two systems crucially interact suggests that considering both systems together might bear a large clinical potential. In line with this idea, both systems have been found altered in PTSD (Yehuda 2002; Neumeister et al. 2013). Most interestingly, the combined analysis of glucocorticoid and endocannabinoid markers was shown to have a higher predictive value for classifying PTSD than the individual analyses (Neumeister et al. 2013). Therefore, pharmacological interventions considering both glucocorticoid- and cannabinoid signaling might be promising and should be further investigated in animal and human models of fear learning and extinction to inform future clinical studies (de Bitencourt et al. 2013).

### Phobias

Glucocorticoid effects on aversive memory processing may not be restricted to traumatic memories in PTSD, but may also include fear memories in phobia. Several studies have investigated the effects of glucocorticoids on fear symptoms in phobic patients. In a randomized controlled trial in 40 patients with social phobia, a single oral dose of cortisone (25 mg) was given 1 h before patients were exposed to the Trier Social Stress Test. As compared to placebo, glucocorticoid treatment significantly reduced stress-induced fear, possibly by affecting memory retrieval processes (Soravia et al. 2006). Importantly, in placebo-treated subjects, the stress-induced release of cortisol was negatively correlated with fear ratings, suggesting that endogenously released cortisol might buffer or counteract fear symptoms in patients with social phobia (Soravia et al. 2006). In another randomized, controlled trial with 20 patients with spider phobia, 10 mg oral cortisol 1 h before the repeated exposure to spider photographs resulted in a gradual reduction of stimulus-induced fear (Soravia et al. 2006). This fear reduction was observed even 2 days after the last drug administration, indicating that glucocorticoids might also have facilitated the extinction of phobic fear.

**Fig. 4:**
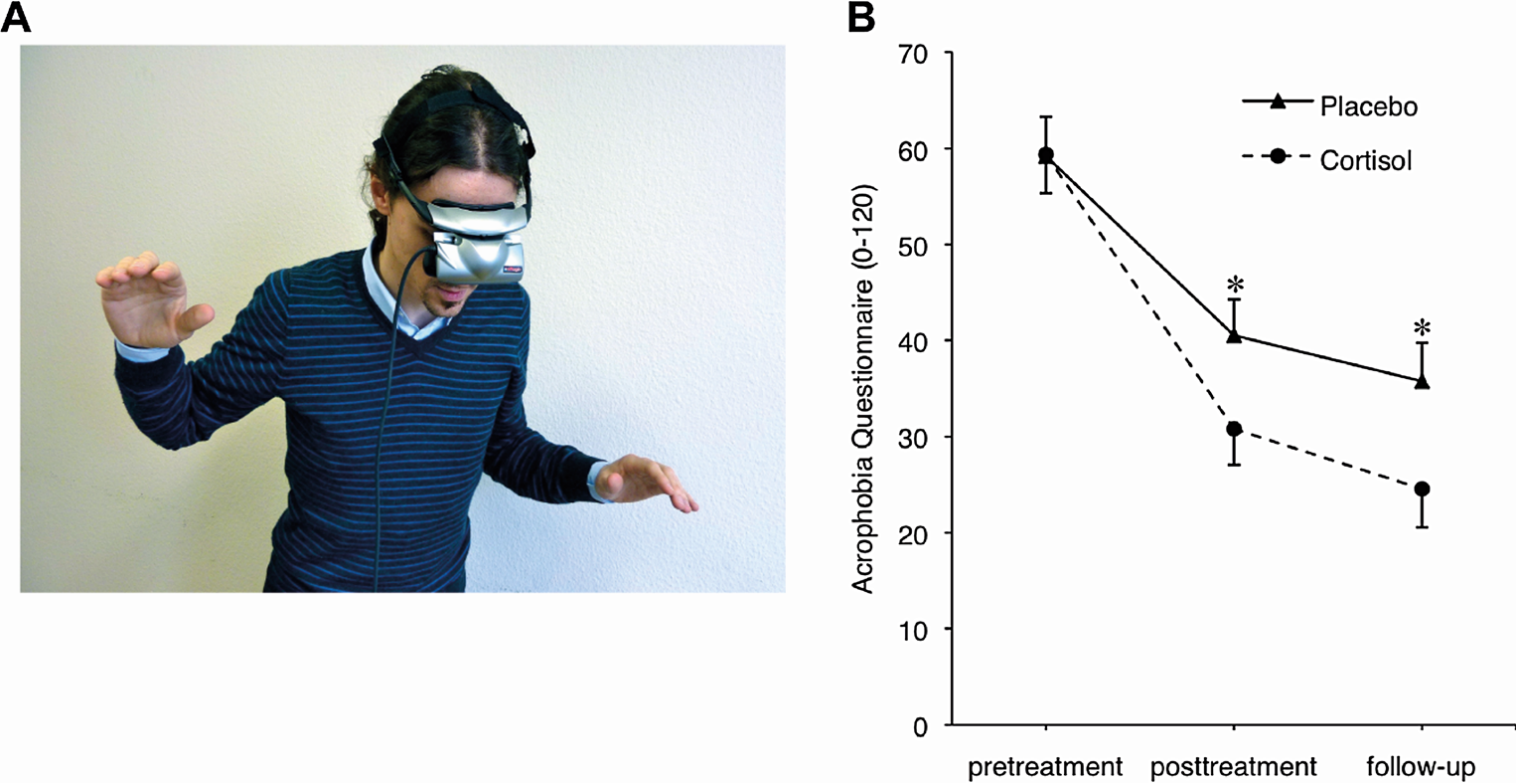
Glucocorticoids enhance extinction-based psychotherapy. **a** VR exposure to fear of heights. **b** Adding cortisol to VR exposure results in reductions of self-reported fear of heights (measured with Acrophobia Questionnaire, range 0–120) at posttreatment and at follow-up. VR exposure took place on 3 treatment sessions between pretreatment and posttreatment assessment. Cortisol (20 mg) was administered 1 h before each VR exposure session. Values are depicted as mean and SEM. Asterisks (*, *P* < 0.05) indicate significant differences between the placebo- and cortisol group at a certain time point. Adapted from (de Quervain et al. 2011)

Because fear extinction is the basis of successful exposure therapy in phobic patients, glucocorticoids might be suited to support this process. One randomized, controlled trial in patients with fear of heights examined whether the administration of glucocorticoids before exposure therapy might enhance treatment outcome (de Quervain et al. 2011). Cortisol (20 mg) or placebo was administered to 40 patients 1 h before each of three virtual-reality exposure sessions. As compared to placebo, cortisol led to a significantly greater reduction of fear of heights at posttreatment and at follow-up (Fig. 4). Moreover, patients receiving the glucocorticoid showed a significantly smaller exposure-induced increase in skin conductance level at follow-up (de Quervain et al. 2011). Another study in subjects with fear of spiders found that the combined administration of cortisol and group exposure therapy enhanced treatment outcome (Soravia et al. 2014). Since these studies indicate that the administration of cortisol has beneficial effects on exposure therapy, also endogenous differences in cortisol levels may have an impact on the outcome of this therapy. This possibility was tested in a study investigating if circadian fluctuations in endogenous cortisol levels affect treatment outcome (Lass-Hennemann and Michael 2014). Patients with fear of spiders who were treated with a single exposure session early in the morning (when cortisol levels are high) showed a significantly greater suppression of fear after treatment than did patients who were treated in the evening (when cortisol levels are low). These effects persisted even at a 3-months follow-up. In line with these findings, a further study showed that time-of-the day-dependent differences in the outcome of exposure therapy are mediated by differences in cortisol levels (Meuret et al. 2016). In conclusion, cortisol is likely to reduce symptoms of phobic fear by reducing aversive memory retrieval and enhancing memory extinction. Therefore, glucocorticoids seem to be well-suited to be administered before extinction-based exposure therapy to support treatment outcome.

### Addiction and other psychiatric disorders

The beneficial effects of glucocorticoids may also expand to other psychopathologies, in which memory, either in symptomatology or therapy, plays a role. In drug addiction, for example, memory plays a role in the storage of associations that provide the powerful incentives for drug taking that produce cravings (Robinson and Berridge 2000; Kelley 2004; Tiffany and Wray 2012; Preller et al. 2013). A recent randomized, controlled trial reported that a single administration of cortisol (20 mg) reduced craving in patients addicted to low-dose heroin (Walter et al. 2015). It is possible that cortisol might have reduced craving by reducing retrieval of addiction memory. However, it has been also reported that the administration of the GR antagonist mifepristone for 1 week decreases alcohol seeking in alcohol-dependent individuals (Vendruscolo et al. 2015).

Furthermore, glucocorticoids might be tested in other psychiatric disorders, in which extinction-based exposure therapy is used, such as in obsessive-compulsive disorder. In this disorder, fear extinction seems to be impaired, which might explain that exposure therapy is difficult and less successful than in phobias (Milad et al. 2013). Therefore, a pharmacological intervention supporting the outcome of exposure therapy of obsessive-compulsive disorder would be highly welcome.

## Conclusions and future perspectives

A wealth of studies has shown that glucocorticoids play a critical role in influencing the consolidation, retrieval, and extinction of emotional memories. Because these memory processes are all highly relevant in the pathogenesis, maintenance and treatment of fear-related disorders, the memory-modulatory properties of glucocorticoids are of considerable translational interest.

Many of the clinical trials reviewed above suggest that the strategy to enhance extinction-based psychotherapy with a timed glucocorticoid administration is a particularly promising approach to treat fear-related disorders. Glucocorticoids may unfold synergistic actions that involve a weakening of dysfunctional memories (through reduced memory retrieval) and a strengthening of psychotherapy-related memories of safety (through enhanced memory extinction). Moreover, several studies have indicated that glucocorticoids may be helpful in preventing the development of PTSD when administered in high doses in the aftermath of a traumatic event.

it is important to note that the existing evidence for the usefulness of glucocorticoids in the prevention and treatment of fear-related disorders comes from rather small proof-of-concept studies. Therefore, large randomized, controlled clinical trials are urgently needed. Furthermore, several open questions should be addressed in future basic and clinical studies. For example, it is still not known what the optimal dosage, time point and duration of glucocorticoid treatment are. Also, safety aspects of such treatments must be assessed in detail. Furthermore, the effects of glucocorticoid administration on context dependency and renewal of exposure therapy have not been investigated in clinical settings.

With regard to dosage, studies investigating PTSD-protective effects of hydrocortisone treatment have used between 20 mg (low dose) and 100 mg (high dose) of cortisol/day administered within 6–12 h after a traumatic event (low dose up to 10 days, high dose 1 to 4 days) (Schelling et al. 2001, 2004; Weis et al. 2006; Zohar et al. 2011; Delahanty et al. 2013). Studies in phobias used single or repeated (up to 4 times) administrations of low doses (10–25 mg) of cortisol (Soravia et al. 2006; de Quervain et al. 2011; Soravia et al. 2014). With regard to safety, potential side effects of glucocorticoids have to be considered. Side effects can typically occur under moderate-to-high dose (30–100 mg) cortisol treatment and the risk increases with prolonged administration or after abrupt offset of such treatments due to suppression of the adrenal response (Stanbury and Graham 1998; Henzen et al. 2000).

It is of great interest to investigate if glucocorticoids have beneficial effects also in other neuropsychiatric disorders, in which memory plays a role in symptomatology or treatment, such as obsessive-compulsive disorder. Further studies might also want to search for epigenetic and genetic markers for diagnostic and/or personalized treatment purposes. Biologically based precision medicine in psychiatry is just beginning to be adopted (Insel and Cuthbert 2015), but such an approach might be indispensable for the identification of patients who are most likely to respond to targeted treatments (Ressler 2018). A recent systemic review provided preliminary evidence that pre-treatment biomarkers, including glucocorticoid sensitivity and metabolism, were able to predict the outcome of psychotherapy of PTSD (Colvonen et al. 2017). Future studies should therefore investigate whether patients with HPA-axis alterations or dysfunctional glucocorticoid signaling might particularly benefit from pharmacological glucocorticoid treatment. Furthermore, basic research might investigate new ways of modulating glucocorticoid signaling to identify more-specific and safe glucocorticoid-related drugs. To conclude, the field of stress and memory research is one of the very few areas in neuroscience where knowledge gained from basic studies have translated into direct clinical applications and it will hopefully continue to do so in the future.
